# The association between land fragmentation and depressive symptoms among Chinese rural households

**DOI:** 10.3389/fpsyg.2026.1792633

**Published:** 2026-05-01

**Authors:** Lei Zhou, Shenwei Wan, Zhiwen Ding, Yong Tang, Liangshan Yang

**Affiliations:** 1 Institute of Rural Development, Zhejiang Academy of Agricultural Sciences, Hangzhou, Zhejiang, China; 2 School of Agricultural Economics and Rural Development, Renmin University of China, Beijing, China; 3 Future Human United Research Institute, Renmin University of China–Westlake University, Beijing, China; 4 Institute of Agricultural Information, Chinese Academy of Agricultural Sciences, Beijing, China

**Keywords:** depressive symptoms, land fragmentation, land transfer, psychological well-being, rural households

## Abstract

**Introduction:**

Land fragmentation is a persistent feature of China’s rural tenure system, yet its psychological ramifications remain underexplored. This study investigates the causal association between land fragmentation and depressive symptoms among rural households, drawing on the Social Determinants of Health framework.

**Methods:**

Using data from the 2022 China Land Economic Survey, we employ an instrumental variable approach based on agricultural machinery subsidies to address endogeneity.

**Results:**

We identify a robust positive impact of fragmentation on depressive symptoms. Mechanism analyses reveal that fragmentation exacerbates depression through three distinct pathways: increasing agricultural production costs, intensifying labor burdens leading to time poverty, and eroding risk coping capabilities by reducing crop insurance participation. Furthermore, land transfer acts as a crucial moderator that significantly attenuates these adverse mental health effects.

**Discussion:**

These findings underscore that land consolidation is not merely an economic imperative but a public health intervention. Policy efforts should integrate land tenure reform with targeted mental health support for vulnerable smallholders.

## 1 Introduction

The global burden of mental disorders constitutes one of the major public health challenges of the 21st century, with depression a leading cause of disability across the world (Vigo et al., 2016; Rehm and Shield, 2019). Although research on urban mental health has proliferated, the psychological wellbeing of rural communities remains critically under-investigated amid rapid agrarian transformations. Crucially, it is essential to distinguish between clinically diagnosed major depressive disorder—which may suffer from significant underreporting in rural areas due to stigma and limited medical resources—and subclinical depressive symptoms. While formal clinical diagnoses might seem less prevalent, subclinical depressive symptoms, such as chronic physical fatigue, emotional exhaustion, and persistent psychological distress, are in fact highly common among smallholder farmers facing severe everyday livelihood friction. One key manifestation of these transformations is land fragmentation, defined as the subdivision of a household’s farmland into many small non-contiguous plots (Tan et al., 2006). In China, this phenomenon stems from the Household Responsibility System and has been intensified by population growth and customary inheritance practices (Yang et al., 2022). Current data reveal that the average farming household cultivates multiple diminutive plots, a reality that constrains agricultural productivity and may have profound implications for farmer wellbeing.

Land occupies a central role in rural livelihoods, serving not only as an economic resource but also as a foundation for social stability, cultural identity and psychological security. When farmland becomes highly subdivided, a cascade of adverse consequences may ensue. Fragmentation undermines efficiency and inflates production costs, placing additional strain on household finances (Tan et al., 2008). It also increases labor demands, engendering greater physical fatigue and acute time scarcity (Tran and Vu, 2019). At the same time, the loss of contiguous land undermines rural households’ sense of control over their environment and livelihoods, contributing to feelings of helplessness and distress (Zhang et al., 2022). As urbanization and land expropriation accelerate, a trend evidenced by the extensive conversion of farmland between 2012 and 2017 (Liu et al., 2022), the health effects of different land tenure patterns emerge as an urgent public health concern.

Despite the potential importance of these issues, empirical research has predominantly focused on the economic implications of land fragmentation while overlooking its psychological ramifications (Aslam and Fazal, 2025). To firmly contextualize our study and refine our core scientific question it is essential to review the existing literature concerning rural poverty land use and mental health. A substantial body of research has extensively investigated the psychological wellbeing of rural populations offering highly valuable insights into the effects of economic deprivation and demographic shifts. For instance, previous studies have rigorously documented the depressive symptoms among rural to urban migrant workers and households facing absolute poverty. Furthermore, concerning land tenure existing scholarship has provided important evidence on the extreme shock of complete land expropriation documenting elevated risks of depression and anxiety among Chinese land lost farmers (Wu J. et al., 2025; Wu R.-Y. et al., 2025). Studies have also identified quantitative investigations of land expropriation and health (Zhao et al., 2022) offering a profound understanding of rural vulnerability. While these foundational studies are indispensable, they tend to conceptualize rural mental health primarily through the lens of quantitative resource deprivation treating land as an economic asset that is either possessed or lost. This conventional paradigm though crucial leaves a potential theoretical space regarding the spatial quality of resource management. The existing literature could benefit from further exploring how the spatial configuration of productive assets affects psychological wellbeing when the total land endowment and household wealth remain largely intact.

Consequently, the core scientific question this study attempts to address is whether the micro structural friction inherent in managing fragmented land constitutes a relevant environmental stressor that operates differently from general economic vulnerability. Land fragmentation might not be treated simply as a proxy for commonly examined economic variables such as income household assets or land loss. Conceptually its psychological consequences differ from both land expropriation and absolute poverty. Land expropriation represents an acute shock of dispossession that can trigger sudden insecurity and identity disruption (Zhao et al., 2022). By contrast fragmentation typically leaves the total land endowment intact yet reshapes the quality of landholding through a dispersed configuration of plots (Lu et al., 2022). This structural distinction is meaningful because households with comparable land area income and asset levels might still face markedly different day to day psychological burdens depending on how their land is spatially organized (Hossain et al., 2022). A specific motivation for our research is to explore the psychological toll of managing agricultural assets inefficiently attempting to distinguish it from the stress of simply having fewer assets.

By exploring this theoretical area our study attempts to introduce an additional explanatory dimension to the literature on rural poverty and mental health which is the spatial organization of agricultural livelihoods. We hope to demonstrate that psychological distress in agrarian settings could be influenced not only by resource scarcity but also by the spatial inefficiency of resource management. As our subsequent empirical analysis attempts to show managing disjointed plots can increase physical labor input and financial costs such as irrigation while potentially reducing formal risk coping capabilities like agricultural insurance. Fragmentation might function as a chronic stressor embedded in routine agricultural life establishing a background of structural hardship (Yueh and Zheng, 2019). The need to meticulously plan across multiple locations monitor heterogeneous soil conditions and synchronize labor across scattered parcels could elevate cognitive load and decision fatigue (Zucchelli et al., 2025). Therefore, we try to conceptualize land fragmentation as a chronic structural stressor that may generate cognitive load financial anxiety and physical exhaustion.

Instead of reiterating general research significance we hope our primary theoretical contribution lies in supplementing the Social Determinants of Health framework. We attempt to shift the academic focus from a purely quantitative assessment of poverty to a qualitative assessment of spatial resource management. By integrating environmental psychology with agricultural economics and by observing the mechanisms of labor constraints financial burdens and institutional vulnerability this study hopes to offer a specific perspective on rural depression. We cautiously hope that our findings will provide useful insights for ongoing rural land reforms aiming to alleviate environmental stressors and improve the mental wellbeing of vulnerable agricultural populations.

## 2 Theoretical logic and research hypothesis

### 2.1 Social determinants of health theory

The theoretical foundation of this study is anchored in the Social Determinants of Health (SDH) framework, as advanced by the World Health Organization. This paradigm posits that health disparities are fundamentally shaped by the conditions in which people are born, grow, live, work, and age, which are in turn influenced by the unequal distribution of power, resources, and money at global, national, and local levels (Irwin et al., 2006; Green, 2010; Chelak and Chakole, 2023). This represents a critical shift from a purely biomedical model of health to a biopsychosocial-environmental model, acknowledging that health outcomes are not merely the product of genetic predisposition or healthcare access but are profoundly embedded in social and economic structures (Bates et al., 2009).

The evolution of this theory reflects a deepening understanding of health determinants. Beginning with the WHO’s 1948 definition of health as “a state of complete physical, mental and social wellbeing”, progressing through the Alma Ata Declaration’s^1^ emphasis on primary healthcare in the 1970s, and culminating in the Commission on Social Determinants of Health’s (CSDH) early-21st-century global goal of “closing the gap in a generation”, this theoretical development highlights a paradigm shift from the biomedical model to a comprehensive biopsychosocial-environmental model. This transformation holds particular significance in the Chinese context, where the “Healthy China 2030” blueprint^2^ explicitly advocates “shifting from a treatment-centered approach to a people-centered health approach,” representing a localized implementation of this conceptual framework.

Drawing on Dahlgren-Whitehead model (Dahlgren and Whitehead, 2021), land fragmentation is conceptualized as a factor operating at the intersection of living and working conditions and the broader socioeconomic environment. It is not merely an agricultural economic phenomenon but a chronic socio-environmental stressor that systematically shapes the material circumstances and psychosocial experiences of rural households. It influences daily routines, economic stability, time allocation, and social interactions, thereby constituting a key element of the “causes of the causes” of health inequities. As research indicates, social factors can trigger a cascade of effects through the perceptual, neuroendocrine, and immune systems, ultimately impacting central nervous system function and mental health (Vukcevic Markovic et al., 2025). We propose that land fragmentation acts as such a stressor, potentially increasing vulnerability to depressive symptoms through multiple pathways.

**Figure 1:**
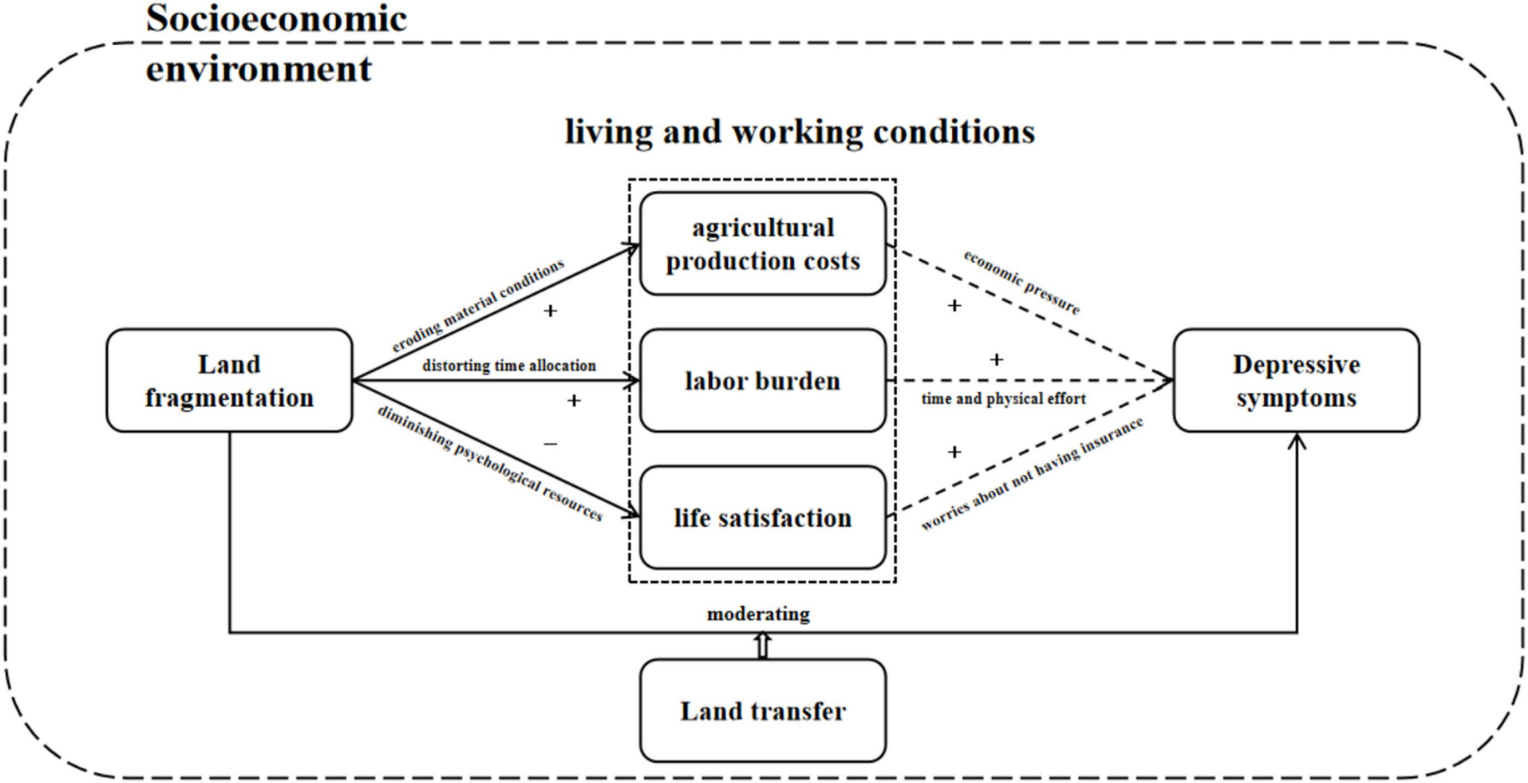
The association pathways of land fragmentation on depressive symptoms via social determinants of health.

Guided by the SDH framework, this study constructs an integrated model (Figure 1) to elucidate the mechanisms linking land fragmentation to depressive symptoms among Chinese rural households. This framework synthesizes concepts from social epidemiology, health economics, and environmental psychology, illustrating a multi-level pathway from macro-level structural factors to micro-level psychological outcomes.

The core logic of the framework is that land fragmentation, as a structural stressor, impacts mental health through three primary mediating pathways: economic pressure, labor burden, and psychosocial distress. These pathways are not mutually exclusive but are likely to be interconnected. The model further incorporates the potential moderating role of adaptive strategies, such as land transfer, and controls for individual and household characteristics. This framework emphasizes that social determinants affect health through the central channel of psychological perception, aligning with the fundamental principle of social medicine. Based on the above analysis, this paper proposes research hypothesis 1.

*H1*: There is an association between land fragmentation and depressive symptoms in Chinese rural households.

### 2.2 Mechanisms and research hypotheses

Grounded in the Social Determinants of Health framework, this study contends that land fragmentation exacerbates depressive symptoms by systematically eroding material conditions, distorting time allocation, and diminishing psychological resources. However, to rigorously test this sophisticated psychological framework using conventional econometric models, it is absolutely essential to establish a robust theoretical bridge between subjective mental states and the observable behavioral proxy variables utilized in our empirical design. We conceptualize that the abstract psychological friction generated by fragmentation is reliably operationalized through three objective socioeconomic pathways. First, the economic pressure pathway captures the severe cognitive burden and persistent financial anxiety arising from the spatial inefficiencies of managing fragmented plots. Instead of relying on potentially biased self-reported measures of subjective financial worry, we utilize agricultural production costs, specifically irrigation and transportation expenses, as the objective behavioral proxy. These elevated operational inefficiencies directly and quantifiably strain household economic stability. Financial insecurity objectively measured by these inflated per mu expenses subsequently fuels persistent anxiety over future livelihoods, activating profound physiological stress responses such as the dysregulation of the hypothalamic pituitary adrenal axis, which fundamentally heightens susceptibility to depression (McCabe and De Judicibus, 2003; Neppl et al., 2021). By logically linking the objective inflation of agricultural production costs to the subjective lived experience of severe economic stress, we bridge the conceptual gap and propose Hypothesis 2.

*H2*: Land fragmentation significantly increases agricultural production costs, which partially mediate its positive association with depressive symptoms.

Labor burden represents a second critical pathway. Managing scattered plots demands excessive time and physical effort, leading to significant time poverty (Chen et al., 2024). This exhaustive labor schedule limits opportunities for restorative leisure activities and social engagement, both of which are vital for maintaining psychological resilience. Physical exhaustion further amplifies stress, creating a cyclical burden that adversely affects mental health (Azzopardi et al., 2019). In rural China, where aging populations and labor migration exacerbate these pressures, the temporal and physical demands of fragmentation become particularly acute. Accordingly, we propose Hypothesis 3.

*H3*: Land fragmentation significantly increases labor burden, which partially mediates its positive association with depressive symptoms.

Risk coping capability forms the third critical pathway. Land fragmentation hinders the adoption of unified management and modern agricultural technologies, increasing exposure to natural and market risks. Fragmented plots are often difficult to insure effectively due to high verification costs and standardized policy limitations, leaving households vulnerable to shocks. The inability to access crop insurance—a key risk management tool—diminishes the household’s sense of security. Persistent worry about uninsured risks and the resulting financial insecurity can directly precipitate anxiety and depressive symptoms (Neppl et al., 2021). Therefore, we propose Hypothesis 4.

*H4*: Land fragmentation significantly reduces household risk coping capability (proxied by crop insurance participation), which mediates its positive association with depressive symptoms.

Beyond these mediating mechanisms, land transfer, the voluntary consolidation or rental of plots could serve as a protective factor. By reducing the direct management burden and providing alternative income through rental payments or opportunities for non-farm employment, land transfer can alleviate economic and labor pressures. It also enhances perceived autonomy, buffering against the psychological distress associated with fragmented landholdings (Ahmed et al., 2021). Policies promoting secure land transfer may thus disrupt the pathway from fragmentation to depression. Accordingly, we propose Hypothesis 5.

*H5*: Land transfer negatively moderates the relationship between land fragmentation and depressive symptoms, with the effect being stronger when the transferred area is larger or involves more consolidated plots.

## 3 Study design and methods

### 3.1 Data sources

The data utilized in this study were drawn from the 2022 China Land Economic Survey (CLES)^3^, a comprehensive survey designed to collect information on land management and economic wellbeing in rural households. The survey employed a Probability Proportional to Size (PPS) sampling method to randomly select sample counties and administrative villages. The research coverage primarily included 13 prefecture-level cities, starting from Jiangsu Province. While Jiangsu is economically developed, it presents a unique context where traditional smallholder fragmentation coexists with rapid agricultural modernization. This tension makes it an ideal setting to observe the psychological costs of the “structural lag” caused by fragmentation. Furthermore, if fragmentation negatively impacts mental health in a relatively prosperous region, the effects are likely more severe in less developed areas, suggesting our findings represent a conservative estimate. All information reported by respondents pertained to their actual circumstances in the year 2021.

**Figure 2:**
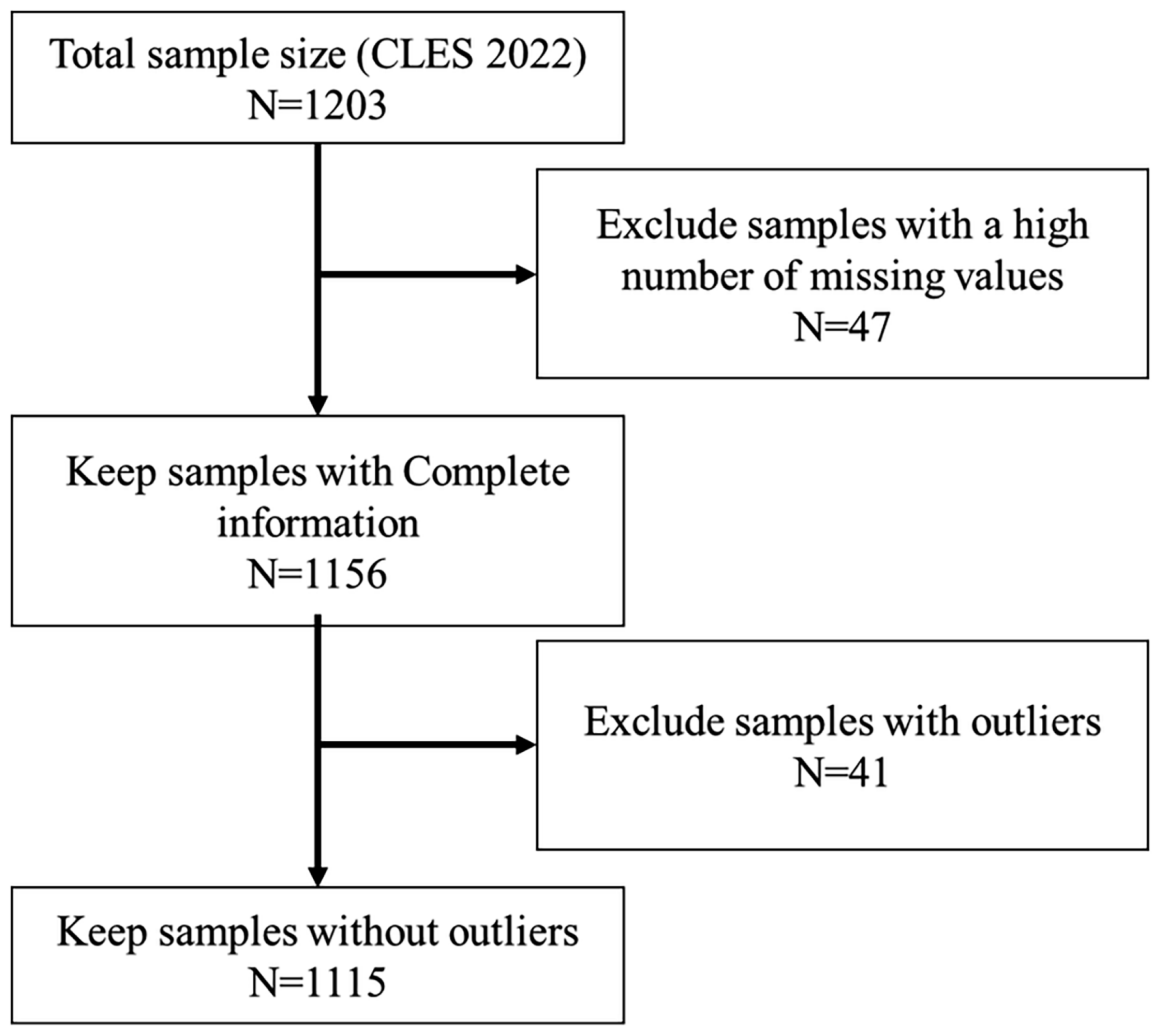
Flowchart of sample selection procedure.

To ensure data quality, a rigorous sample selection process was implemented, as illustrated in Figure 2. The initial sample from the CLES 2022 comprised 1,203 households. We first excluded 47 samples with a high proportion of missing values. From the remaining 1,156 households with complete information, we further identified and excluded 41 samples containing significant outliers. Consequently, the final analytical sample consisted of 1,115 household observations. The sample reflects the prevalence of small-scale farming in the region, with 47.89% of households managing a total land area of less than 1 mu^4^, highlighting the dominance of smallholders in our study.

The selection of Jiangsu Province as our empirical setting serves a specific methodological purpose regarding external validity. Although Jiangsu is an economically advanced province with relatively high rates of land transfer and large-scale agricultural operations, it paradoxically faces exceptionally high population-land pressure. According to official data from the Third National Land Survey and the Seventh National Population Census, the national average per capita cultivated land is approximately 1.36 mu. In contrast, Jiangsu’s massive resident population results in a per capita cultivated land of merely 0.73 mu. This severe human-land tension dictates that a vast number of smallholder farmers in Jiangsu must still operate on highly constrained and fragmented plots.

Therefore, rather than undermining our study, this context provides a conservative stress test for our theoretical framework. In less developed regions, it is often difficult to distinguish whether a farmer’s depression is caused by land fragmentation itself or simply by absolute poverty. The Jiangsu sample helps isolate this confounding factor. If land fragmentation operates as a psychological stressor even in a wealthy region with advanced social safety nets, it suggests that the spatial friction of fragmentation possesses independent explanatory power. Consequently, the negative psychological effects observed in this relatively affluent sample likely represent a lower-bound estimate. This strongly indicates that mental health burdens derived from spatial inefficiency could be substantially more severe in less developed agricultural regions, thereby supporting the broader generalizability of our findings.

### 3.2 Variable selection

#### 3.2.1 Assessment of the depressive symptoms of rural households

The dependent variable in this study is the depression index (*CESD8*) of rural residents, which is calculated by summing the scores of eight mental health-related questions. As shown in Table 1, the scale consists of eight items assessing the frequency of various emotions and behaviors during the past week. Each item is scored on a 1–4 point scale, with the total score ranging from 8 to 32. Higher scores indicate more severe depressive symptoms, while lower scores suggest milder symptoms. It should be explicitly noted that the CES-D 8 scale is designed to evaluate a continuum of psychological distress and subclinical depressive symptoms—such as chronic fatigue, emotional exhaustion, and sleep disturbance—rather than to serve as a diagnostic tool for clinical major depressive disorder. Therefore, our dependent variable captures the varying degrees of everyday psychological friction and chronic stress experienced by farmers, reflecting a widespread subclinical mental health burden rather than the prevalence of psychiatric diseases.

**Table 1:** Depression index (CESD8) measurement scale.

Item code	Measurement content	Scoring criteria	Scoring method
K2-01	I felt depressed	1 = Rarely (less than 1 day) 2 = Sometimes (1–2 days) 3 = Often (3–4 days) 4 = Most of the time (5–7 days)	Positive scoring
K2-02	I felt everything was an effort		Positive scoring
K2-03	My sleep was restless		Positive scoring
K2-04	I was happy		Reverse scoring
K2-05	I felt lonely		Positive scoring
K2-06	I enjoyed life		Reverse scoring
K2-07	I felt sad		Positive scoring
K2-08	I felt I could not go on		Positive scoring

This measurement instrument demonstrates good reliability and validity and has been widely used in relevant domestic and international studies (Chen et al., 2026; Sun et al., 2024; Wu J. et al., 2025). Specifically, validity studies have confirmed that the CES-D 8 maintains high internal consistency and construct validity among Chinese rural elderly populations, making it suitable for this study’s context.

#### 3.2.2 Assessment of the land fragmentation

The core explanatory variable is the degree of land fragmentation (LF). We operationalize this using the inverse of the average plot size. First, the average plot size is calculated by dividing the total cultivated area ( Total Area) by the number of plots ( Number of Plots) . To intuitively interpret fragmentation (where smaller plots equal higher fragmentation), we take the reciprocal of this value. To mitigate the influence of outliers often produced by inverse transformations, the variable was winsorized at the 1 and 99% levels during data cleaning. This operationalization is chosen to better capture the actual operational friction experienced by farmers. While the absolute number of plots intuitively reflects land division, incorporating the total area accounts for the crucial dimension of spatial density. For example, managing 5 plots spread across 10 mu involves different management intensity and physical constraints compared to managing 5 plots confined to just 1 mu. By using the inverse of the average plot size, this index standardizes the measurement to reflect relative spatial inefficiency. This approach helps quantify the microscopic size of parcels that can restrict mechanization and generate daily physical and cognitive stress, offering a more nuanced metric than an absolute count of land parcels. Equation 1:


LF=1/(Total Area/Number of Plots) (1)

#### 3.2.3 Assessment of mediating variables

Three key mediating variables were operationalized to examine the pathways through which land fragmentation influences depressive symptoms. Labor input per mu (days/mu), denoted as *labor*, captures the intensity of household labor expenditure in agricultural production. Irrigation cost per mu (yuan/mu), labeled as *cost*, reflects the economic burden associated with water management across fragmented plots. Crop insurance expenditure (yuan), indicated as *insurance*, represents risk mitigation behaviors adopted by households facing production uncertainties. These variables collectively illuminate the mechanistic pathways linking land fragmentation to mental health outcomes.

#### 3.2.4 Assessment of moderator variables

In practice, rural households often engage in land transfer as a strategic approach to mitigate the adverse effects of land fragmentation. Through transferring out land parcels, households can effectively reduce labor inputs and production costs associated with fragmented plots, while simultaneously generating rental income and divesting from less suitable land. This adaptive behavior positions land transfer as a significant moderator in the relationship between land fragmentation and rural households’ mental health outcomes. To quantitatively assess this moderating effect, the study employs two key indicators: the total area of transferred land (*Area_transferred*) and the number of transferred plots (*Plots_transferred*). These metrics capture different dimensions of land transfer behavior, with the former reflecting the scale of transfer and the latter indicating the consolidation effect on land fragmentation patterns.

#### 3.2.5 Assessment of covariates

To account for potential confounding factors in examining the relationship between land fragmentation and rural households’ mental health, this study incorporates control variables from two primary dimensions: individual characteristics and household attributes, based on established theoretical frameworks and empirical literature.

At the individual level, we control for the following variables: respondent’s age (*age*), measured in years; gender (*gender*), coded as 1 for male and 0 for female; education level (*edu*), recorded as years of formal schooling; and health status (*health*), assessed using a five-point Likert scale. These variables account for fundamental individual-level characteristics that may influence mental health outcomes. Regarding household characteristics, three key variables are included: number of agricultural laborers in the household (*laborers*), referring to family members primarily engaged in farming; total household expenditure (*total_exp*), encompassing annual spending on both production operations and living consumption; and household savings (*savings*), indicating the family’s financial buffer capacity. These covariates control for the potential effects of household resource endowments on psychological wellbeing.

### 3.3 Model setting

To empirically examine the association of land fragmentation on rural households’ depressive symptoms, we first establish the following baseline regression model (Equation 2):


$$\text{CESD}8_{\mathrm{it}}=\beta_{0}+\beta_{1}{\mathrm{LF}}_{\mathrm{it}}+\beta^{\prime }C\mathrm{on}\text{trol}s_{\mathrm{it}}+\varepsilon_{\mathrm{it}}$$ (2)

Here, the dependent variable $\text{CESD}8_{\mathrm{it}}$ represents the depression index of household *i* in year *t*, measured using the CESD8 scale with higher scores indicating more severe depressive symptoms. The core explanatory variable ${\mathrm{LF}}_{\mathrm{it}}$ ​ denotes the degree of land fragmentation, calculated as the reciprocal of the average plot area. A higher value indicates greater fragmentation. $C\mathrm{on}\text{trol}s_{\mathrm{it}}$ ​ comprises a set of control variables including individual characteristics and household attributes. $\varepsilon_{\mathrm{it}}$ is the idiosyncratic error term.

The model is estimated using ordinary least squares (OLS). To ensure the validity of our estimates, we conducted multicollinearity tests, with all variance inflation factors (VIF) below 10, indicating no severe multicollinearity concerns. Furthermore, we cluster standard errors to account for potential heteroskedasticity.

## 4 Results and analysis

### 4.1 Descriptive statistical analysis

Table 2 presents descriptive statistics for all variables in this study, based on a sample of 1,115 household observations. The dependent variable, depressive symptoms (*CESD8*), shows a mean of 14.5846 with a standard deviation of 2.2606, indicating moderate variation in depressive symptoms among sampled households. The core explanatory variable, land fragmentation (*LF*), has a mean of 0.9792 and standard deviation of 1.3134, suggesting substantial heterogeneity in fragmentation levels across households.

**Table 2:** Descriptive statistics.

Type	Variable	Definition	Mean	Std. Dev.	Min	Max
Explained variables	CESD8	depressive symptoms	14.5846	2.2606	8.0000	26.0000
Core explanatory variables	LF	Degree of fine fragmentation of land	0.9792	1.3134	0.0125	10.0000
Mediator variables	Labor	Self-invested labor input man-days per mu	34.4972	55.1192	0.0000	360.0000
	Cost	Irrigation cost per mu	72.3193	96.9389	0.0000	750.0000
	Insurance	Planting insurance	486.2284	1973.6160	0.0000	14000.0000
Moderator variables	Area transferred	Cultivated land transferred out (mu)	3.0373	3.4777	0.0000	16.0000
	Plots transferred	Cultivated land transferred out of blocks	2.7787	2.0207	0.0000	12.0000
Covariate variables	Age	Age of respondent	62.2465	11.1610	30.0000	81.0000
	Gender	Gender of respondent	0.7368	0.4406	0.0000	1.0000
	Edu	Years of education	7.0752	4.1731	0.0000	16.0000
	Health	Health level of the respondent	3.9568	1.0856	1.0000	5.0000
	Laborers	Number of agricultural laborers in the household	2.4845	1.0063	1.0000	5.0000
	Total_exp	Total household expenditure	40400.0000	43300.0000	3400.0000	275000.0000
	Savings	Household savings	39900.0000	69300.0000	0.0000	400000.0000

Regarding mediator variables, self-invested labor input (*labor*) demonstrates significant variation with a mean of 34.4972 and standard deviation of 55.1192. Irrigation cost per mu (*cost*) shows a mean of 72.3193 and standard deviation of 96.9389, reflecting uneven distribution of production costs. The exceptionally high standard deviation (1973.6160) for crop insurance expenditure (*insurance*) indicates vast differences in households’ insurance participation behaviors.

For moderator variables, the mean values of transferred land area (3.0373 mu) and number of transferred plots (2.7787) suggest widespread participation in land transfer among sampled households. Control variables reveal that respondents averaged 62.25 years of age, were predominantly male (73.68%), had 7.08 years of formal education on average, and reported generally good health (mean 3.96/5). Household characteristics indicate an average of 2.48 agricultural laborers per household, with annual total expenditure averaging 40,400 yuan and savings averaging 39,900 yuan.

### 4.2 Benchmark regression analysis

As shown in Table 3, this study examines the association of land fragmentation on rural households’ depressive symptoms through three progressive models. Model (1) containing only the core explanatory variable shows a coefficient of 0.2386 for land fragmentation, significant at the 1% level, providing preliminary evidence of a positive correlation. After incorporating individual characteristics in Model (2), the coefficient for land fragmentation remains stable (0.2295, significant at 1%), while gender, education level and health status demonstrate significant negative effects on depressive symptoms. Model (3) further controlling for household characteristics maintains the significant positive coefficient for land fragmentation (0.2321, significant at 1%), with individual characteristics retaining their significance and direction, though household characteristics show no statistical significance. Therefore, Hypothesis 1 is supported.

**Table 3:** Analysis of baseline regression results.

Variables	Model (1)	Model (2)	Model (3)
	CESD8	CESD8	CESD8
LF	0.2386***	0.2295***	0.2321***
	(0.0667)	(0.0661)	(0.0671)
Age		−0.0156	−0.0143
		(0.0095)	(0.0097)
Gender		−0.4015*	−0.4177*
		(0.2114)	(0.2147)
Edu		−0.0486*	−0.0458*
		(0.0249)	(0.0253)
Health		−0.2983***	−0.2953***
		(0.0915)	(0.0927)
Laborers			0.0660
			(0.1069)
Total_exp			0.0000
			(0.0000)
Savings			−0.0000
			(0.0000)
Constant	14.2267***	17.0646***	16.7610***
	(0.1092)	(0.8092)	(0.8837)
*R*-squared	0.021	0.058	0.059

* Means *p* < 0.1, ** means *p* < 0.05, *** means *p* < 0.01, and the standard errors in parentheses are the same below.

Notably, the coefficient variation for land fragmentation across the three models is less than 0.01, indicating robust effects on depressive symptoms. The R-squared value increases progressively from 0.021 in Model (1) to 0.059 in Model (3), suggesting enhanced explanatory power with the inclusion of control variables. The constant term demonstrates 1% significance across all models with reasonable numerical variations.

### 4.3 Addressing endogeneity

To address potential endogeneity concerns between land fragmentation and rural households’ depressive symptoms, this study employs an instrumental variable (IV) approach. We select “agricultural machinery purchase subsidy amount” (*subsidy*) as the instrument, which satisfies both relevance and exclusion conditions. Mechanization, land, Regarding relevance subsidies directly incentivize mechanization, which technically requires consolidated land, thus driving households to proactively reduce fragmentation (Deininger et al., 2017). Regarding the crucial exclusion restriction, we must systematically and transparently address potential confounding macro level channels such as local governance capacity, regional development conditions, and household wealth effects. First, the agricultural machinery purchase subsidy is fundamentally a national unified public policy implemented with exceptionally strict physical criteria across all jurisdictions. The subsidy allocation is rigidly determined by the specific model and horsepower of the machinery purchased rather than by the discretionary local public service quality, the financial agility of county governments, or the personal social capital networks of the applying farmers, thereby substantially mitigating the risk of unobserved macroeconomic confounding. Second, concerning the valid possibility that subsidies might directly alleviate general economic stress and consequently improve psychological wellbeing, we emphasize that this specific policy operates primarily as a post-purchase financial reimbursement rather than an unconditional liquid cash transfer. It mandates that households first endure significant initial capital investment and effectively does not immediately relax daily consumption budget constraints for the family. Furthermore, our rigorous empirical models explicitly control for total household expenditure and aggregate savings, effectively absorbing background variations in general economic stress and subjective psychological security. Consequently, the primary and most theoretically sound residual pathway through which this earmarked physical capital subsidy influences rural mental health remains via compelling the spatial consolidation of farmland to accommodate large-scale mechanization, thereby directly neutralizing the structural stressor of fragmentation.

Table 4 presents the two-stage least squares (2SLS) estimation results. The first-stage regression shows a significantly negative coefficient for the instrument *subsidy*, consistent with theoretical expectations that machinery subsidies reduce fragmentation. The second-stage results confirm a persistently significant positive effect of land fragmentation on depressive symptoms after controlling for endogeneity, validating the robustness of our baseline findings. The first-stage F-statistic was 28.45, exceeding the threshold of 10, which rejects the null hypothesis of weak instruments. The first-stage R^2^ of 0.479 indicates strong explanatory power of the instrument. The consistency in significance levels and coefficient directions with baseline models further reinforces the reliability of our conclusions.

**Table 4:** Results of endogeneity test (Two-stage IV).

Variables	Model (4)	Model (5)
	First	Second
	LF	CESD8
LF		1.3959***
		(0.4160)
Subsidy	−0.0002***	
	(0.0000)	
Age	0.0102*	−0.0269
	(0.0055)	(0.0183)
Gender	0.0448	−0.2027
	(0.0622)	(0.1976)
Edu	0.0037	−0.0380*
	(0.0061)	(0.0222)
Health	0.0279	−0.1513*
	(0.0265)	(0.0807)
Laborers	−0.1237***	0.0750
	(0.0402)	(0.1364)
Total_exp	0.0000	−0.0000
	(0.0000)	(0.0000)
Savings	−0.0000**	−0.0000
	(0.0000)	(0.0000)
Constant	0.3131***	16.0557***
	(0.4267)	(1.5531)
*R*-squared	0.479	0.025

* means *p* < 0.1, ** means *p* < 0.05, *** means *p* < 0.01, and the standard errors in parentheses are the same below.

### 4.4 Robustness check

To verify the reliability of research findings, this study conducts robustness checks through variable replacement and dimension decomposition strategies as shown in Table 5. Models (6) through (15) systematically examine the impact of land fragmentation on rural households’ psychological wellbeing under different variable specifications.

**Table 5:** Robustness check: variable replacement and dimension decomposition.

Variables	Model (6)	Model (7)	Model (8)	Model (9)	Model (10)	Model (11)	Model (12)	Model (13)	Model (14)	Model (15)
	Replace explanatory variables	Replace the explained variable	CESD8 items							
	CESD8	Life satisfaction	K2-01	K2-02	K2-03	K2-04	K2-05	K2-06	K2-07	K2-08
LF		−0.2134*	0.0412**	0.0370**	0.0616**	0.0524*	0.0409***	0.0021***	0.0465***	0.0298***
		(0.1259)	(0.0165)	(0.0165)	(0.0247)	(0.0294)	(0.0079)	(0.0008)	(0.0146)	(0.0091)
Xy1m	0.0093** (0.0039)									
Number of plots	0.0821* (0.0475)									
Age		0.0465	−0.0081***	−0.0040	−0.0118***	0.0082	−0.0054	0.0061*	−0.0028	−0.0036**
		(0.0354)	(0.0026)	(0.0026)	(0.0039)	(0.0084)	(0.0181)	(0.0031)	(0.0023)	(0.0014)
Gender		−1.7814***	−0.1278**	−0.1745***	−0.1175	0.1854	0.2488	0.1583**	−0.1126**	−0.0570*
		(0.4731)	(0.0583)	(0.0585)	(0.0875)	(0.1154)	(0.1677)	(0.0702)	(0.0518)	(0.0323)
Edu		0.1797**	−0.0085	−0.0120*	−0.0153	0.0021	−0.0224	0.0099	−0.0089	−0.0089**
		(0.0824)	(0.0069)	(0.0069)	(0.0104)	(0.0110)	(0.0254)	(0.0083)	(0.0061)	(0.0038)
Health		0.1101	−0.1011***	−0.1116***	−0.1957***	0.1379***	−0.0337	0.1438***	−0.0895***	−0.0455***
		(0.2621)	(0.0251)	(0.0251)	(0.0376)	(0.0414)	(0.1033)	(0.0302)	(0.0222)	(0.0139)
Laborers		−0.7383***	−0.0581**	−0.0350	0.0187	0.1319***	−0.0899	0.0988***	−0.0181	−0.0051
		(0.2436)	(0.0289)	(0.0290)	(0.0434)	(0.0482)	(0.0894)	(0.0348)	(0.0256)	(0.0160)
Total_exp		0.0000	0.0000*	0.0000***	0.0000	−0.0000*	0.0000	−0.0000***	0.0000***	0.0000
		(0.0000)	(0.0000)	(0.0000)	(0.0000)	(0.0000)	(0.0000)	(0.0000)	(0.0000)	(0.0000)
Savings		−0.0000*	−0.0000**	−0.0000	−0.0000	0.0000	−0.0000	0.0000***	−0.0000**	−0.0000
		(0.0000)	(0.0000)	(0.0000)	(0.0000)	(0.0000)	(0.0000)	(0.0000)	(0.0000)	(0.0000)
Constant		6.6500**	2.4741***	2.2122***	3.1621***	1.7175***	−0.0054	0.0061*	1.8373***	1.5687***
		(2.6095)	(0.2379)	(0.2387)	(0.3571)	(0.6177)	(0.0181)	(0.0031)	(0.2112)	(0.1318)
*R*-squared		0.116	0.085	0.096	0.073	0.087	0.479	0.025	0.099	0.066

* means *p* < 0.1, ** means *p* < 0.05, *** means *p* < 0.01, and the standard errors in parentheses are the same below.

Model (6) further verifies the reliability of the research findings by sequentially employing two alternative explanatory variables to replace the original land fragmentation degree. First, we utilize the “number of plots below 1 mu”. This substitution captures the micro-characteristics of fragmentation from the perspective of land size distribution, avoiding potential linear assumption limitations of continuous variables. The empirical results show a significantly positive coefficient at the 5% level, indicating that a higher concentration of extremely small parcels stably predicts worse mental health outcomes. Second, we introduce the “absolute number of plots” (without adjusting for total land area) as another alternative measure to capture the pure physical division of farmland. The regression results indicate that this absolute plot count also exerts a positive effect on depressive symptoms, remaining statistically significant at the 10% level. By interpreting these results together, the consistent positive significance across these two distinct measurement dimensions provides rich empirical support, confirming the robustness of the adverse effects of land fragmentation on the psychological wellbeing of rural households across different measurement approaches.

Model (7) substitutes the dependent variable by using life satisfaction (1–10 scale) instead of the depression index. This transformation validates relationship stability from a positive psychology perspective, with a significantly negative coefficient indicating that fragmentation not only exacerbates psychological distress but directly undermines subjective wellbeing, expanding the theoretical implications of impact effects.

Model (8)-Model (15) implement dimension decomposition by regressing each of the CESD8 items as independent dependent variables. Results demonstrate stronger effects on core symptoms like “depressed mood” and “everything being an effort,” while reverse-scored items such as “feeling happy” show weaker associations. This symptom specificity suggests fragmentation primarily intensifies negative affect rather than diminishing positive emotions, providing nuanced evidence for understanding mental health mechanisms under land institution influences.

## 5 Discussion

### 5.1 Mechanism of action analysis

This study substantiates the Social Determinants of Health framework by identifying land tenure structure specifically the spatial organization of agricultural production as a critical environmental determinant of mental wellbeing. Existing sociological and economic studies have traditionally attributed rural depressive symptoms to absolute economic deprivation lower social status or acute shocks such as land expropriation. Unlike previous studies focusing primarily on economic outcomes (Nguyen et al., 1996) our mechanism analysis attempts to introduce a fundamentally new explanatory dimension to the literature on rural poverty and mental health. We provide robust empirical evidence demonstrating that the structural inefficiency of land layout generates a distinct form of psychological friction operating independently of the households total wealth. This approach shifts the academic focus from a purely quantitative assessment of poverty to a qualitative assessment of spatial resource management. We attempt to establish that land fragmentation is not merely a barrier to economic productivity but a chronic and embedded environmental stressor. We explicitly examine the specific behavioral pathways through which this spatial stressor affects rural household’s depressive symptoms using rigorous mediation effect models. As shown in Table 6 Models 16 through 18 present the regression results treating labor input irrigation costs and insurance expenditure as mediating variables, respectively.

**Table 6:** Mechanism analysis results.

Variables	Model (16)	Model (17)	Model (18)
	Labor	Cost	Insurance
LF	38.3250*	52.4128*	−213.5173***
	(22.3220)	(29.4155)	(79.1599)
Age	0.2844	−1.4534	−28.2319*
	(1.1136)	(1.7225)	(15.9573)
Gender	23.6143	−17.4558	778.1465**
	(17.1744)	(37.2381)	(334.8277)
Edu	1.6383	0.3755	−46.3268*
	(1.9211)	(3.8550)	(25.7667)
Health	−21.3586*	−21.6831	293.5489***
	(11.0744)	(14.7467)	(112.7987)
Laborers	2.7562	−14.1186	339.2992*
	(6.6283)	(14.7509)	(186.4725)
Total_exp	0.0005	0.0006	0.0164***
	(0.0004)	(0.0004)	(0.0053)
Savings	−0.0001	−0.0003**	0.0020
	(0.0001)	(0.0001)	(0.0022)
Constant	38.5110	298.9343**	−376.9202
	(101.8791)	(148.3603)	(1,099.3250)
*R*-squared	0.069	0.050	0.095

* means *p* < 0.1, ** means *p* < 0.05, *** means *p* < 0.01, and the standard errors in parentheses are the same below.

Regarding the labor input mechanism Model 16 demonstrates that a one unit increase in land fragmentation leads to an additional 38.3250 labor days per mu which is statistically significant at the 10 percent level. Traditional rural poverty literature often focuses on severe unemployment or low wage physical labor as primary stressors but our finding highlights a completely different structural burden. Rather than simply interpreting this coefficient as a routine numerical reallocation of daily working hours as standard economic models might we attempt to understand the profound physiological and temporal toll it represents. Managing widely dispersed plots imposes severe temporal constraints and deep somatic poverty upon the household. In the inescapable context of an aging rural demographic this severe spatial inefficiency forces elderly farmers to substitute intense physical human exertion for mechanized capital simply because large machines cannot access small divided parcels (Tran and Vu, 2019). This mandatory extra labor of over 38 days per mu leads inevitably to chronic biological exhaustion and physical deterioration serving as direct physiological precursors to depressive symptoms. This clarifies how spatial mismanagement independently produces physical stress confirming our second hypothesis.

Furthermore, the irrigation cost mechanism tested in Model 17 shows a significantly positive effect of land fragmentation on irrigation costs per mu at the 10 percent level yielding a substantial coefficient of 52.4128. In conventional agricultural economic research this specific finding might simply be viewed as a routine accounting loss on a ledger or a basic reduction in profit margins. However, advancing our new spatial environmental perspective this empirical finding captures the immense psychological weight of persistent financial friction. An additional cost of 52 yuan per mu represents an enduring and exhaustive cognitive burden where vulnerable farmers constantly struggle to allocate severely scarce financial resources across disjointed parcels. Every time they irrigate a fragmented plot they are reminded of this operational penalty fueling a continuous state of chronic anxiety and daily economic dread that gradually erodes their overall mental wellbeing. This distinct financial anxiety is derived entirely from spatial inefficiency rather than absolute income loss which robustly supports our third hypothesis.

Finally, Model 18 presents the risk coping mechanism analysis where land fragmentation exerts a significantly negative impact on crop insurance expenditure with a coefficient of negative 213.5173 significant at the 1 percent level. Existing literature usually attributes low insurance participation among smallholder farmers to a lack of financial awareness or insufficient premium subsidies. In contrast our spatial perspective reveals a profound and deeply rooted institutional vulnerability. Because highly fragmented and miniscule plots face severe verification barriers and exorbitant administrative costs in formal insurance markets this specific spatial configuration structurally strips smallholder households of their most critical formal risk coping mechanisms. This systemic institutional exclusion leaves farmers perilously exposed to unpredictable natural disasters and extreme weather fluctuations. The inability to buy a basic safety net breeds a pervasive sense of helplessness loss of perceived life control and long term developmental insecurity. This completely aligns with the psychosocial stress etiology proposed in our overarching theoretical framework highlighting a novel pathway where spatial structure dictates institutional protection and thereby validating our fourth hypothesis. In addition among control variables health status shows a significantly negative effect in the labor input equation indicating better health reduces perceived labor burden while household savings significantly negatively affect irrigation costs reflecting the moderating role of economic buffering capacity.

### 5.2 Analysis of moderating effects

Before directly analyzing the regulatory effect of land transfer it is crucial to position this behavior within our newly proposed spatial environmental framework. Table 7 presents the split sample analysis results based on land transfer status. For households without land transfer Model 19 indicates that land fragmentation maintains a significant positive effect on depressive symptoms with a coefficient of 0.2199 at the 1 percent level. In stark contrast for households participating in land transfer Model 20 shows that the coefficient decreases to 0.1782 and becomes statistically insignificant. Traditional agricultural economic literature typically conceptualizes land transfer strictly as a market mechanism designed to achieve economies of scale and optimize resource allocation. However, interpreting this statistical divergence through our spatial psychological perspective reveals a fundamentally different explanatory dimension. We attempt to demonstrate that land transfer serves as a proactive spatial coping strategy for vulnerable households. By selectively transferring out distant or highly disjointed parcels farmers can effectively dismantle the physical and cognitive barriers that generate spatial stress thereby actively mitigating the adverse psychological effects of fragmentation which strongly supports our fifth hypothesis.

**Table 7:** Heterogeneity of whether land is transferred out.

Variables	Model (19)	Model (20)
	No land transfer	Land transfer
	CESD8	CESD8
LF	0.2199***	0.1782
	(0.0779)	(0.1412)
Age	0.0032	−0.0203*
	(0.0160)	(0.0114)
Gender	−1.4823***	0.0955
	(0.3847)	(0.2404)
Edu	−0.0016	−0.0531*
	(0.0431)	(0.0289)
Health	−0.4190***	−0.1540
	(0.1571)	(0.1043)
Laborers	−0.1519	0.1886
	(0.1758)	(0.1293)
Total_exp	0.0000***	−0.0000
	(0.0000)	(0.0000)
Savings	−0.0000	−0.0000
	(0.0000)	(0.0000)
Constant	16.9693***	16.0175***
	(1.4214)	(1.0337)
*R*-squared	0.201	0.025

* means *p* < 0.1, ** means *p* < 0.05, *** means *p* < 0.01, and the standard errors in parentheses are the same below.

Table 8 further examines this buffering mechanism through the incorporation of interaction terms. Model 21 demonstrates a significantly negative coefficient of negative 0.0436 for the interaction between fragmentation and transferred area at the 10 percent level. Similarly Model 22 reveals a significantly negative coefficient of negative 0.0214 for the interaction with the number of transferred plots. The economic significance of these moderating effects is profound indicating that for each additional mu of transferred land the impact of fragmentation on depressive symptoms decreases by approximately 15.8 percent. Correspondingly each additional transferred plot reduces the psychological effect by about 7.4 percent. Conventional studies often interpret such reductions merely as the result of increased rental income alleviating absolute poverty. In contrast our theoretical framework argues that these highly significant negative interaction terms confirm the moderating role of land transfer in physically attenuating the fragmentation and depression relationship by restoring a sense of environmental control and perceived autonomy (Ahmed et al., 2021). When farmers successfully consolidate their operational space by shedding the most inefficient plots, they experience a massive reduction in daily cognitive load and physical exhaustion.

**Table 8:** Moderating role of land transferred out.

Variables	Model (21)	Model (22)
	CESD8	CESD8
LF	0.2765***	0.2909**
	(0.0858)	(0.1269)
LF*Area_ transferred	−0.0436*	
	(0.0415)	
LF* Plots_ transferred		−0.0214*
		(0.0319)
Control variables	YES	YES
Constant	16.3922***	16.5639***
	(1.5205)	(1.4489)
*R*-squared	0.147	0.137

* means *p* < 0.1, ** means *p* < 0.05, *** means *p* < 0.01, and the standard errors in parentheses are the same below.

These moderating findings deeply enrich our dialogue with the existing literature on rural mental health and poverty. They provide compelling empirical support for our core argument that the psychological toll of agriculture is profoundly dictated by the spatial organization of livelihoods rather than just the total amount of household assets. We attempt to establish that land transfer policies should no longer be viewed solely as economic productivity interventions but must be fundamentally recognized as highly effective spatial public health interventions. By facilitating the reconfiguration of disjointed landholdings these policies empower farmers to actively alter their stressful physical environments. This synthesis explicitly answers the call for a new explanatory dimension showing that rural depression can be mitigated not just by injecting financial capital but by optimizing the spatial structure of daily agricultural operations to improve rural households psychological wellbeing.

### 5.3 Heterogeneity analysis

Building upon our mechanism analysis we attempt to further explore the boundary conditions of this spatial psychological stress process through a detailed heterogeneity analysis based on land tenure certification status and skills training backgrounds. Table 9 presents the six sets of regression results evaluating these heterogeneities. Regarding the institutional environment the outcomes demonstrate that formal land certification significantly buffers the negative psychological impacts of fragmentation. Specifically, the coefficient of land fragmentation is 0.2670 and significant at the 1 percent level in uncertified households which is notably higher than the 0.2084 observed in certified households. Traditional institutional economics often views land certification primarily as an administrative mechanism to secure economic property rights and facilitate market transactions. However, when interpreted through our newly proposed spatial environmental perspective this statistical disparity suggests that legal tenure security functions as a critical psychological safety net (Bui et al., 2020). By providing stable legal expectations and mitigating fears of arbitrary land reallocation certification dramatically reduces the anticipatory anxiety of potential land disputes typically associated with vague and highly fragmented spatial boundaries. It effectively alleviates the overall cognitive load on farmers illustrating how institutional clarity can directly buffer spatial stress.

**Table 9:** Heterogeneity analysis results.

Variables	Model (23)	Model (24)	Model (25)	Model (26)	Model (27)	Model (28)
	Land certification	No land certification	Non-agricultural vocational education	No non-agricultural vocational education	Agriculturally trained	No agriculturally trained
LF	0.2084**	0.2670***	0.1613	0.2571***	0.2216	0.2370***
	(0.0966)	(0.0736)	(0.1147)	(0.0672)	(0.2138)	(0.0619)
Age	−0.0260*	−0.0082	−0.0036	−0.0181	−0.0003	−0.0175*
	(0.0145)	(0.0121)	(0.0147)	(0.0115)	(0.0331)	(0.0098)
Gender	−0.1171	−0.6606**	−0.1535	−0.4038*	0.5830	−0.4525**
	(0.3762)	(0.2610)	(0.4567)	(0.2392)	(0.6759)	(0.2247)
Edu	−0.0969**	−0.0168	−0.0362	−0.0421	0.0100	−0.0630**
	(0.0410)	(0.0318)	(0.0499)	(0.0286)	(0.0831)	(0.0270)
Health	−0.1098	−0.3996***	−0.1847	−0.2894***	−0.7157*	−0.2292**
	(0.1601)	(0.1089)	(0.1752)	(0.1022)	(0.3628)	(0.0941)
Laborers	0.2135	−0.0709	0.2193	−0.0019	0.8458**	−0.0283
	(0.1520)	(0.1411)	(0.1762)	(0.1221)	(0.3847)	(0.1093)
Total_exp	−0.0000	0.0000**	0.0000	0.0000	0.0000	0.0000
	(0.0000)	(0.0000)	(0.0000)	(0.0000)	(0.0000)	(0.0000)
Savings	−0.0000	−0.0000	−0.0000	−0.0000	0.0000	−0.0000
	(0.0000)	(0.0000)	(0.0000)	(0.0000)	(0.0000)	(0.0000)
Constant	16.7918***	16.7859***	15.0369***	17.0244***	14.2242***	17.0839***
	(1.4611)	(1.0863)	(1.5632)	(1.0146)	(3.0802)	(0.8963)
*R*-squared	0.053	0.114	0.076	0.068	0.187	0.077

* means *p* < 0.1, ** means *p* < 0.05, *** means *p* < 0.01, and the standard errors in parentheses are the same below.

Furthermore, the human capital analysis reveals a highly significant moderating effect that complements our theoretical framework. The coefficient on farmland fragmentation was positive and significant at the 1 percent level at 0.2571 for the sample without nonagricultural vocational education while it dropped to a statistically insignificant level for those with such education. Similarly, the coefficient is significant at 0.2370 for those without agricultural training but becomes insignificant for their trained counterparts. Existing literature on rural poverty reduction typically conceptualizes vocational training solely as an economic tool for income generation and absolute poverty alleviation. In contrast our spatial psychological perspective interprets this compelling empirical evidence as an indication that diversified human capital endows farmers with broader livelihood resilience and superior cognitive flexibility (Talukdar et al., 2020). When farmers acquire modern skills they gain diverse ways to make a living allowing them to psychologically detach their core self-worth and ultimate economic security from the immediate daily inefficiencies of fragmented farming.

These heterogeneity findings provide a concrete and nuanced synthesis of our theoretical dialogue with the existing literature. Rather than merely proving that vulnerable farmers suffer more our results systematically demonstrate how spatial friction interacts with social structures. We attempt to show that the severe mental health deterioration experienced by farmers lacking both institutional legal protection and modern skill sets is not an inevitable biological consequence of farming but a socially constructed vulnerability. By integrating these moderating factors, we advance the Social Determinants of Health framework by illustrating a multi layered stress process. We establish that the psychological burden of managing disjointed plots is an environmental stressor that can be actively neutralized by macro level institutional policies and micro level educational interventions. This effectively introduces a new theoretical dimension showing that rural depression stems from the complex intersection of spatial inefficiency institutional exclusion and human capital constraints rather than simple economic deprivation.

## 6 Limitations

This study has several limitations that warrant consideration. First, the cross-sectional nature of the data limits our ability to strictly establish causal directionality. Although we employed sophisticated instrumental variable techniques using agricultural machinery purchase subsidies to mitigate potential endogeneity, we must openly acknowledge that establishing a flawless exclusion restriction relies substantially on profound theoretical argumentation rather than exhaustive empirical elimination. Despite controlling for major household economic confounders, we cannot entirely and definitively rule out the possibility that the spatial distribution and intensity of such governmental subsidies might residually correlate with unobserved systemic regional factors like local governance efficiency comprehensive rural development initiatives or localized social support networks which could independently and simultaneously foster psychological wellbeing among farmers. Moreover, substantial financial subsidies might still exert subtle long-term wealth effects or fundamentally alter household future livelihood expectations through multifaceted channels operating parallel to and distinct from land consolidation. Given the inherent absence of alternative viable instrumental variables within the survey data to perform formal overidentification tests, the ultimate strength of our strict causal inference is inherently bounded by these observational constraints. The compelling statistical associations documented comprehensively herein should therefore be prudently interpreted as highly robust structural relationships shedding light on spatial inequalities rather than absolute definitive causal proofs necessitating future rigorous validation through extensive longitudinal panel designs. Second, regarding the mechanisms, while we identified risk coping (insurance) as a pathway, reciprocal causation is possible—depressive symptoms might arguably reduce a household’s proactive engagement in insurance markets. Longitudinal data would be required to disentangle these temporal dynamics. Third, the sample was drawn exclusively from Jiangsu Province. While this region provides a critical window into the tension between fragmentation and modernization, the findings may not be fully generalizable to less developed agricultural regions in Western China, where the psychological buffering resources might differ. Future research should incorporate multi-regional panel data to enhance external validity and causal inference. Fourth, the operationalization of land fragmentation inherently influences the magnitude of the observed statistical effects. Our study utilizes the inverse of average plot size to emphasize spatial density and management friction. However, as with any unidimensional metric, this measurement approach might highlight specific structural disparities. As a potential boundary condition, if fragmentation were measured strictly by the absolute number of plots without accounting for total area scale, the statistical disparity and the magnitude of the psychological impact might be less pronounced, as an absolute count does not fully capture the intense physical constraints on mechanization and cost-efficiency. Future research should strive to employ multidimensional fragmentation indices—incorporating precise spatial distance, plot shape, and distribution indices—to comprehensively untangle the nuances of these spatial stressors.

## 7 Conclusion

This study systematically investigates the relationship between land fragmentation and depressive symptoms among rural residents in China, drawing on data from the 2022 China Land Economic Survey. The evidence demonstrates that fragmentation of farmland constitutes a potent structural stressor in agrarian settings, with consistently detrimental effects on psychological wellbeing.

Empirical models reveal a robust positive association between the degree of land fragmentation and subclinical depressive symptomatology (manifesting as everyday psychological distress and cognitive fatigue). This structural stressor persists after adjusting for individual demographic factors and household socioeconomic status, highlighting a widespread but often overlooked subclinical mental health burden among smallholders, rather than an epidemic of clinical psychiatric disorders. Quantitatively, a one-mu reduction in average plot size (approximately 0·067 ha) corresponds to an increase of 0·32 points in the depressive symptom score (*β* = 0·32, 95%CI:0·18–0·46; *p* < 0·01). Mediation analyses identify three principal pathways: fragmentation raises per-unit production costs by 15–20%, particularly through elevated irrigation expenses; it extends labor input time by 25–30%, engendering severe time poverty; and it undermines psychosocial wellbeing via eroded risk coping capabilities (reduced insurance participation), leaving households vulnerable to shocks. Moderation analysis further shows that participation in land transfer attenuates the effect of fragmentation on depressive symptoms by approximately 40% (β reduction from 0.41 to 0.25), suggesting that reconfiguring landholdings can buffer adverse psychological impacts.

On the basis of these profound empirical insights, our policy implications must radically transcend conventional agricultural directives like simply promoting market-driven land transfer or blindly increasing machinery subsidies. The fundamental theoretical contribution of this comprehensive study lies in decisively expanding the etiology of rural depression well beyond conventional absolute poverty narratives to formally and rigorously incorporate the spatial organization of agricultural livelihoods. By empirically demonstrating that the structural misallocation of land resources generates distinct and potent psychological friction entirely independent of total household wealth, we introduce a critically new explanatory dimension to rural mental health research that bridges environmental psychology and agricultural economics.

Therefore, at the macro policy level, state-led land consolidation programs must be fundamentally reconceptualized not merely as economic productivity tools, but as vital proactive spatial public health interventions. To precisely address the unhealthy socio-psychological issues arising from rural land systems, we propose three well-targeted and actionable solutions:

First, policymakers should actively promote’ operational consolidation’ through subsidized agricultural socialized services. By encouraging professional cooperatives to provide outsourced services (such as mechanized plowing, planting, and harvesting) for fragmented plots, smallholders can delegate the severe physical and cognitive burdens to professionals. This actionable strategy achieves economies of scale in daily operations without forcing vulnerable farmers to formally transfer their land rights, thereby protecting their fundamental sense of security.

Second, local administrations should pioneer grassroots’ cooperative spatial swapping’ (Hu-huan-bing-di) mechanisms. Instead of pushing for rapid, top-down land expropriation or mandatory transfers that might strip farmers of their emotional attachment to the land, village committees can facilitate voluntary plot exchanges among neighboring farmers. This highly actionable approach allows households to naturally consolidate their operational space and eliminate physical barriers, while keeping their total land endowment completely intact.

Third, the government must proactively embed localized mental health support directly into existing rural agricultural networks. Specifically, grassroots agricultural extension workers should be systematically retrained to not only provide farming technical assistance but also conduct baseline psychological stress assessments. By utilizing this pre-existing, trusted rural network, mental health interventions can precisely target aging smallholders deeply trapped in the structural lag between rigid traditional land tenure and modern agricultural demands.

By treating agricultural spatial efficiency as a foundational prerequisite for rural health equity, governments can fundamentally disrupt the hidden pathological pathway from structural environmental stress to individual psychological deterioration, delivering highly targeted and transformative welfare improvements to the most vulnerable agrarian populations.

To strengthen causal inference, longitudinal cohort studies are needed to track the evolution of depressive symptoms in relation to changing land-use patterns. Cross-cultural comparisons could elucidate how different tenure systems moderate fragmentation’s mental health effects, while incorporation of physiological stress markers and neuroimaging techniques may further clarify the biopsychosocial mechanisms at work.

## Data Availability

The original contributions presented in the study are included in the article/supplementary material, further inquiries can be directed to the corresponding authors.

## Footnotes

1 Declaration of Alma-Ata (https://www.who.int/teams/social-determinants-of-health/declaration-of-alma-ata)

2
https://www.who.int/teams/health-promotion/enhanced-wellbeing/ninth-global-conference/healthy-china

3
http://agbigdata.aiijournal.com/CN/10.19788/j.issn.2096-6369.230418

4 Mu is a unit of measurement for land area in China. One hectare is equivalent to 15 mu.
